# Efficacy of autologous cell therapy on limb salvage in patients with chronic limb-threatening ischemia: 16-year single-center experience

**DOI:** 10.1186/s13287-025-04493-1

**Published:** 2025-07-15

**Authors:** Dominika Sojakova, Michal Kahle, Jitka Husakova, Vladimira Fejfarova, Karol Sutoris, Radka Jarosikova, Edward B. Jude, Michal Dubsky

**Affiliations:** 1 Diabetes Centre, Institute for Clinical and Experimental Medicine, Prague, Czech Republic; 2 First Faculty of Medicine, Charles University, Prague, Czech Republic; 3 Department of Data Science, Institute for Clinical and Experimental Medicine, Prague, Czech Republic; 4 Transplantation Surgery Department, Institute for Clinical and Experimental Medicine, Prague, Czech Republic; 5 Diabetes Center, Tameside and Glossop Integrated Care NHS Foundation Trust, Ashton-under-Lyne and University of Manchester, Manchester, UK

**Keywords:** Stem cell therapy, Peripheral artery disease, Ischemia, Amputation, Amputation-free survival

## Abstract

**Backgrounds:**

Autologous cell therapy (ACT) could be a treatment option for patients with chronic limb-threatening ischemia (CLTI) when standard vascular intervention is impossible. This study aimed to analyze risk factors affecting therapeutic success and identify patients with diabetes most responsive to ACT.

**Methods:**

In this prospective study, 129 treatments were provided to 118 limbs in 107 no-option CLTI patients with diabetes. Bone marrow was obtained, and stem cells were processed and injected into the calf muscles of the affected limb. After 16 years, we analyzed the influence of baseline factors related to patients (diabetes parameters, comorbidities, medications), limb ischemia (TcPO_2_ value, Graziani and GLASS classifications), ulcer (descriptions according to Wagner, WIfI, SINBAD and Texas classifications), and infection (the value of CRP, the presence of the osteomyelitis, resistant bacteria and clinical signs of infections). Outcomes were limb salvage (LS) and amputation-free survival (AFS), which were assessed using Cox regression models.

**Results:**

Major amputation was performed in 41 out of 118 limbs (31.8%). The use of immunosuppressive therapy (HR 2.48, CI 1.30–4.73), higher stages of GLASS FP (femoropopliteal) score (HR 1.58, CI 1.31–1.90) in the univariate model, and signs of clinical infection (HR 2.21, CI 1.01–4.839) in the multivariable model significantly impacted LS. Shorter AFS was associated with a higher GLASS FP score (HR 1.28, CI 1.13–1.46), dialysis (HR 2.05, CI 1.33 − 3.16 ), hypoalbuminemia (HR 0.93, CI 0.89–0.98), signs of clinical infection (HR 1.99, CI 1.26–3.15) in the univariable model, and immunosuppression (HR 2.31, CI 1.09–4.95) in the multivariable model.

**Conclusion:**

Decisions to manage patients with no-option CLTI should be based on involvement of the peripheral circulation, the presence of infection and co-morbidities. Those with minimal impairment of the FP segment, with the best possible nutritional status and without signs of infection would benefit the most. Furthermore, we should be careful with dialysis patients and those on immunosuppressive therapy.

## Introduction

Peripheral artery disease (PAD) and neuropathy are the leading causes of diabetic foot disease, resulting in foot ulcerations and limb loss. PAD prevalence is about 5% at 40–44 years of age and rises to around 12% at 70–74 years, predominantly in people with diabetes, in whom PAD is two– seven -fold more prevalent than in people without it [1, 2]. Chronic limb-threatening ischemia (CLTI) is a clinical syndrome defined by the presence of PAD in combination with rest pain, gangrene or foot ulcer of at least 2 weeks duration [3]. There are several strategies for the treatment of CLTI– endovascular revascularization (percutaneous transluminal angioplasty– PTA) and surgical methods including bypass and endarterectomies.

However, approximately 25% of CLTI patients are not eligible for standard revascularization methods (no-option CLTI) [4]. Patients who are not candidates for revascularization have minimal chances of avoiding major amputation, and their mortality is very high. The risk of limb loss in no-option CLTI is 20–25% at 1 year after receiving a diagnosis of CLTI [5]. For these no-option patients, autologous stem cell therapy (ACT) could be an alternative approach. The most common precursor cells used for this therapy are bone marrow mononuclear cells (BM-MNCs), peripheral blood mononuclear cells (PB-MNCs) after prior stimulation with granulocyte-colony stimulating factor (G-CSF) or granulocyte-macrophage colony-stimulating factor (GM-CSF), and mesenchymal stem/stromal cells (MSCs), which can be isolated from various tissues [6].

We have been performing BM-MNC cell therapy at our center for 16 years. The aim of this study was to analyze pre-selected risk factors that could identify the patients that would benefit the most from this therapy.

As the endpoints of ACT success, we have chosen limb salvage (LS) and amputation-free survival (AFS). While LS means only time without necessity of a major amputation, AFS refers to survival time without major amputation or death from any cause. Both are also used in other studies which assess outcomes of patients after vascular surgery, CLTI patients without any treatment or efficacy of cell therapy in no-option CLTI patients [5, 7–9].

## Materials and methods

### Study population

This is a longitudinal prospective study including patients with diabetes and CLTI without the option of standard revascularization. Study inclusion criteria were: diagnosis of diabetes, age 18–90 years, presence of CLTI with ulcers or gangrene proven by transcutaneous oxygen pressure (TcPO_2_) below 30 mmHg or ischemic pain lasting at least 2 weeks. Moreover, as we noted above, in these patients there was no option for standard revascularization (PTA, bypass or endarterectomy). Exclusion criteria were the presence of signs of systemic infection, deep foot infection with phlegmona, severe edema of the limb, severe hematological abnormalities, deep vein thrombosis, myocardial infarction or stroke in the last 6 months, acute untreated severe diabetic retinopathy, diagnosed tumor of any organ, and inability to undergo general anesthesia. Ineligibility for standard revascularization procedures was always decided by a multidisciplinary team consisting of diabetologists, interventional radiologists, and vascular surgeons. After evaluating all inclusion and exclusion criteria, eligible patients underwent standard blood tests (full blood count, C-reactive protein (CRP), creatinine), standard cancer screening (chest X-ray, abdomen ultrasound, tumor markers, fecal occult blood test, PSA level in men, and mammogram in women), and an eye fundus examination. If there were no contraindications after these examinations, we proceeded with pre-screening, which included X-rays of the affected limb; infection antibodies (hepatitis panel, HIV and syphilis testing); coagulation profile; complete blood count; biochemistry (renal and liver parameters, mineral analysis, albumin, lipid profile and Hb1Ac); and CRP.

### ACT therapy

The actual procedure of ACT involved the harvesting of 250 ml of bone marrow using a Jamshidi needle, and the bone marrow was then mixed with a heparin solution. Stem cell products were isolated using two different methods in accordance with recent European Medicines Agency guidelines and the Czech State Institute for Drug Control. In the first method (*n* = 93), bone marrow was filtered, injected into four 60 mL centrifugation containers, and centrifuged at 4000 rpm (revolutions per minute) using the Harvest Smart PReP2 (Harvest Technologies Corporation, Plymouth, MA, USA). The second method was based on filtering bone marrow with Gelofusine solution and centrifuging it using a benchtop centrifuge (Hettich ROTANTA 460R, Andreas Hettich GmbH & Co. KG Germany) (*n* = 36). The reason for the change in the method used for cell separation was the impossibility of further purchase components for the Harvest method. After the isolation, the product was injected intramuscularly into the calf of the patient by a surgeon in the operating room on the same day as the bone marrow trephine biopsy. Each cell product underwent microbiological monitoring in accordance with Good Laboratory Practice.

### Study schedule

After 16 years of the treatment program, we assessed baseline risk factors and their impact on LS and AFS. LS is an outcome that refers to the duration without requiring a major amputation in which the death of the patient is a censoring event. In contrast, AFS is defined as the survival without major amputation and death from any cause. Therefore it includes both death and major amputation as the endpoint. All baseline factors with characteristics of patients, hematological and immunohistochemical parameters of the final product injected into the treated limb, are presented in Table 1. Patient-related risk factors included sex, age, diabetes parameters (type of diabetes and duration, diabetes treatment and Hb1Ac), status of nutrition (BMI, serum albumin, lipid profile), the presence of comorbidities (stage of chronic kidney disease, neuropathy, eye complications, coronary artery disease and heart failure the latter defined by echocardiography and NT-proBNP or BNP levels), and medication history (statins, antithrombotic drugs, and immunosuppression for any reason).

**Table 1:** Baseline characteristics of the patients and limbs at the time of treatment

Characteristic		
Female		17/129 (13%)
Age [years]		67.23 ± 10.04
Smoking	Yes	15/129 (12%)
	Ex	58/129 (45%)
	No	56/129 (43%)
**Diabetes**		
Type	1.	18/129 (14%)
	2.	104/129 (81%)
	Secondary	7/129 ( 5%)
Duration [years]		21.00 (12.00, 28.00)
HbA1c [mmol/mol]		58.37 ± 14.97
Therapy	Diet	6/129 ( 5%)
	Oral antidiabetcs	33/129 (26%)
	Insulin	86/129 (67%)
	Pancreas transplant	4/129 ( 3%)
**Complications**		
Chronic kidney disease	1	39/129 (30%)
	2	35/129 (27%)
	3	28/129 (22%)
	4	5/129 ( 4%)
	5	22/129 (17%)
Hemodialysis		22/129 (17%)
Eye complications		75/129 (58%)
Neuropathy		96/129 (74%)
Coronary artery disease		89/129 (69%)
Heart Failure		42/129 (33%)
**Nutrition and lipids**		
BMI [kg/m2]		27.45 ± 4.14
Albumin [g/L]		35.78 ± 5.52
Cholesterol [mmol/L]		3.92 ± 1.03
LDL [mmol/L]		2.18 ± 0.87
TAG [mmol/L]		1.55 (1.09, 2.27)
**Medication**		
Statins		75/129 (58%)
Immunosuppression		28/129 (22%)
Antithrombotic drugs	No	1/129 ( 1%)
	ASA	87/129 (67%)
	P2Y12 inhibitors	27/129 (21%)
	Warfarin	15/129 (12%)
	LMWH	18/129 (14%)
	DOAC	16/129 (12%)
**Ischemia**		
TcPO_2_ [mmHg]		19.00 (8.00, 30.00)
Rutherford classification	4	3/129 ( 2%)
	5	116/129 (90%)
	6	10/129 ( 8%)
Graziani classification	3	6/129 ( 5%)
	4	38/129 (29%)
	5	50/129 (39%)
	6	30/129 (23%)
	7	4/129 ( 3%)
Glass FP	0	39/129 (30%)
	1	34/129 (26%)
	2	18/129 (14%)
	3	13/129 (10%)
	4	20/129 (16%)
Glass IP	1	2/129 ( 2%)
	2	4/129 ( 3%)
	3	40/129 (31%)
	4	78/129 (60%)
Glass P	0	12/129 ( 9%)
	1	70/129 (54%)
	2	42/129 (33%)
**DFU classifications**		
Wagner	1	5/129 ( 4%)
	2	40/129 (31%)
	3	20/129 (16%)
	4	64/129 (50%)
Wifi Infection	0	61/129 (47%)
	1	43/129 (33%)
	2	23/129 (18%)
	3	2/129 ( 2%)
Wifi Ischemia	1	2/129 ( 2%)
	2	6/129 ( 5%)
	3	121/129 (94%)
Wifi Wound	0	1/129 ( 1%)
	1	24/129 (19%)
	2	92/129 (71%)
	3	12/129 ( 9%)
Wifi Composite	1	1/129 ( 1%)
	3	1/129 ( 1%)
	4	18/129 (14%)
	5	47/129 (36%)
	6	38/129 (29%)
	7	15/129 (12%)
	8	9/129 ( 7%)
Texas Grade	1	14/129 (11%)
	2	85/129 (66%)
	3	30/129 (23%)
Texas Stage	C	91/129 (71%)
	D	38/129 (29%)
Sinbad	0	1/129 ( 1%)
	2	6/129 ( 5%)
	3	35/129 (27%)
	4	53/129 (41%)
	5	26/129 (20%)
	6	8/129 ( 6%)
**Ulcer infection**		
Clinical infection		42/129 (33%)
CRP [mg/l]		5.60 (2.20, 15.00)
OM X-ray signs		45/122 (37%)
MRSA		14/129 (11%)
Resistant bacteria		58/129 (45%)
**Isolation method**		
Harvest		93/129 (72%)
Gelofusin		36/129 (28%)
**Final product**		
Leukocytes [10^9/L]		43.30 (29.80, 58.40)
Lymphocytes [10^9/L]		8.00 (5.20, 12.85)
Monocytes [10^9/L]		1.73 (1.10, 2.87)
Thrombocytes [10^9/L]		292.00 (154.50, 455.50)
CD34_percentage [%]		0.52 (0.37, 0.77)
CD34_count [10^6/l]		198.83 (115.79, 368.26)
Viability [%]		96.00 (93.70, 98.20)

Abbreviation:

DM - diabetes mellitus, BMI - body mass index, ASA - aspirin, LMWH - low-molecular-weight heparin, DOAC - non-vitamin-K-antagonist oral anticoagulants, TcPO2 - transcutaneous oxygen pressure, GLASS - Global Limb Anatomic Staging System, FP - femoropopliteal segment, IP– infrapopliteal segment, P - pedal segment, DFU - diabetic foot ulcer, OM– osteomyelitis, MRSA - Methicillin-resistant Staphylococcus aureus

To assess the limb ischemia we used TcPO_2_, Rutherford category [10], Graziani stages [11], and The Global Limb Anatomic Staging System (GLASS) containing the stenosis assessment in the femoropopliteal (FP), infrapopliteal (IP) and pedal (P) segment [12]. This classification is used for prediction of revascularization success following the patency of the target artery pathway (TAP) [13]. TAP was defined by the ulcer location. For wounds on the dorsum of the foot, TAP is the anterior tibial artery, which supplies the dorsalis pedis artery, for wounds on the plantar surface of the foot, the TAP is the posterior tibial artery, which supplies the plantar circulation through the medial and lateral plantar arteries and the plantar arch. Finally, for lateral foot wounds or heel ulcers, the TAP is the peroneal artery, which provides collateral supply to the foot when other pathways are compromised.

Ulcer healing was evaluated using several classifications including Wagner, TEXAS [14], WIfI, and SINBAD classifications [15]. In addition, the WIfI composite score, encompassing elements of WIfI classifications including description of wound, ischemia and infection, was also added to our study. Clinically, signs of infection according to standard Virchow features were observed, such as redness, swelling, warmth and signs of systemic inflammation. However the pain symptoms could be masked by diabetic peripheral neuropathy. CRP was used as a laboratory marker of infection. Osteomyelitis was assessed by X-ray. Resistant bacteria were recorded in the microbial findings of the ulcers.

Finally, parameters of the final ACT product, specifically leukocytes, monocytes, lymphocytes, product viability, total number of CD34 subpopulation and percentage of these subpopulations were included in this study.

Discontinuation of follow-up could occur for three reasons: major amputation, defined as any resection proximal to the ankle, death, or treatment failure, which was defined by the value of TcPO_2_ below 30 mmHg in the third month of follow-up. In the latter, the patients could receive stem cells a second time in the same limb and were re-enrolled in the ACT study with new measured baseline parameters.

### Statistical analysis

Data are shown as mean ± SD or median (1st quartile– 3rd quartile). All confidence intervals (CI) span 95%. The effects of predictors on survival were assessed by Cox proportional hazards regression. The proportional hazards assumption was tested by scaled Schoenfeld residuals. No correction for multiple testing was performed. The follow-up was truncated on 1 March 2025. All calculations were performed using the Python ecosystem and the Lifelines package [16]. The dataset with analysis methods used in the study is available at [https://doi.org/10.5281/zenodo.15111180] [17].

## Results

**Fig. 1:**
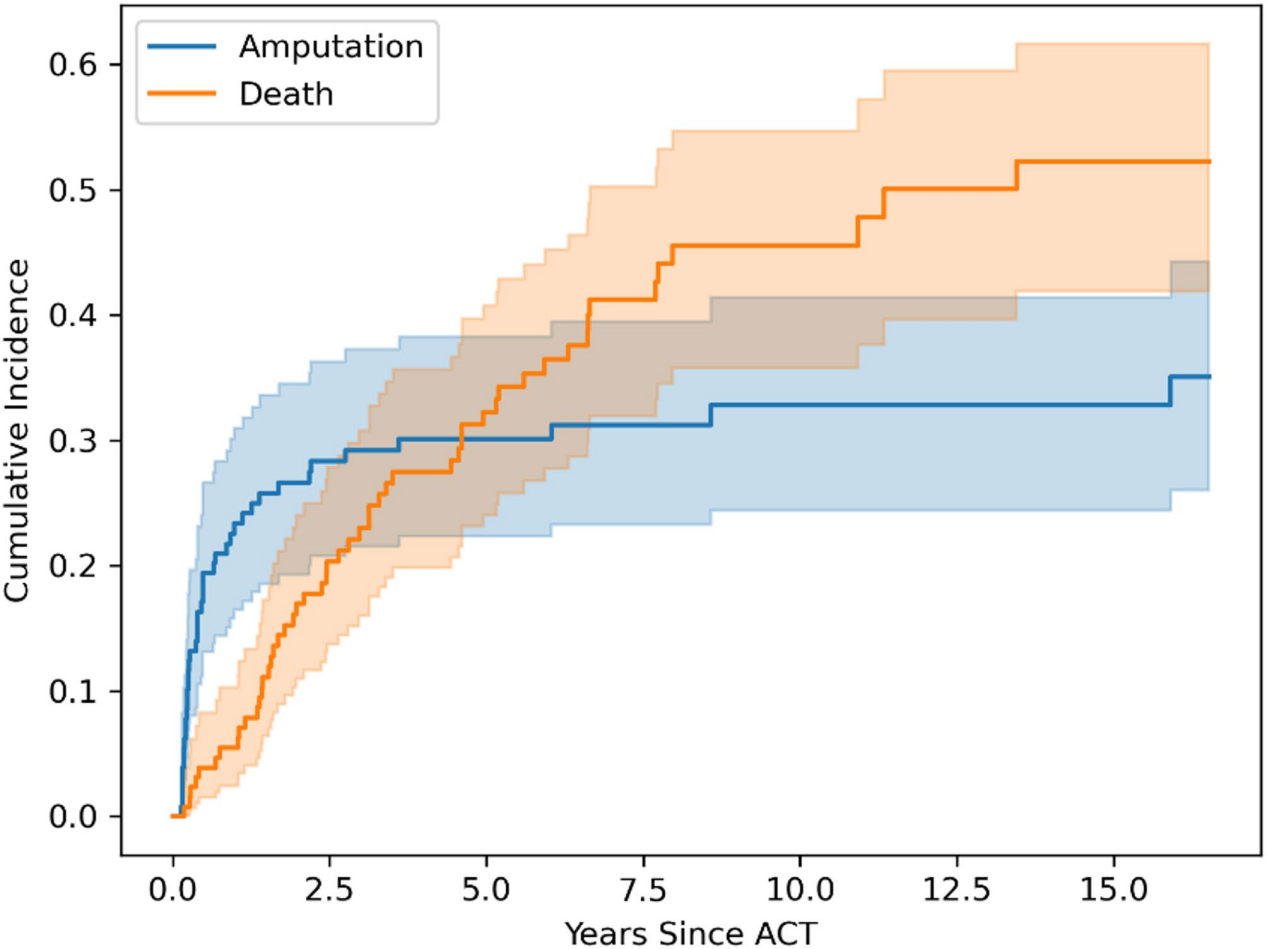
Cumulative incidence of major amputations and death during ACT follow up

**Fig. 2:**
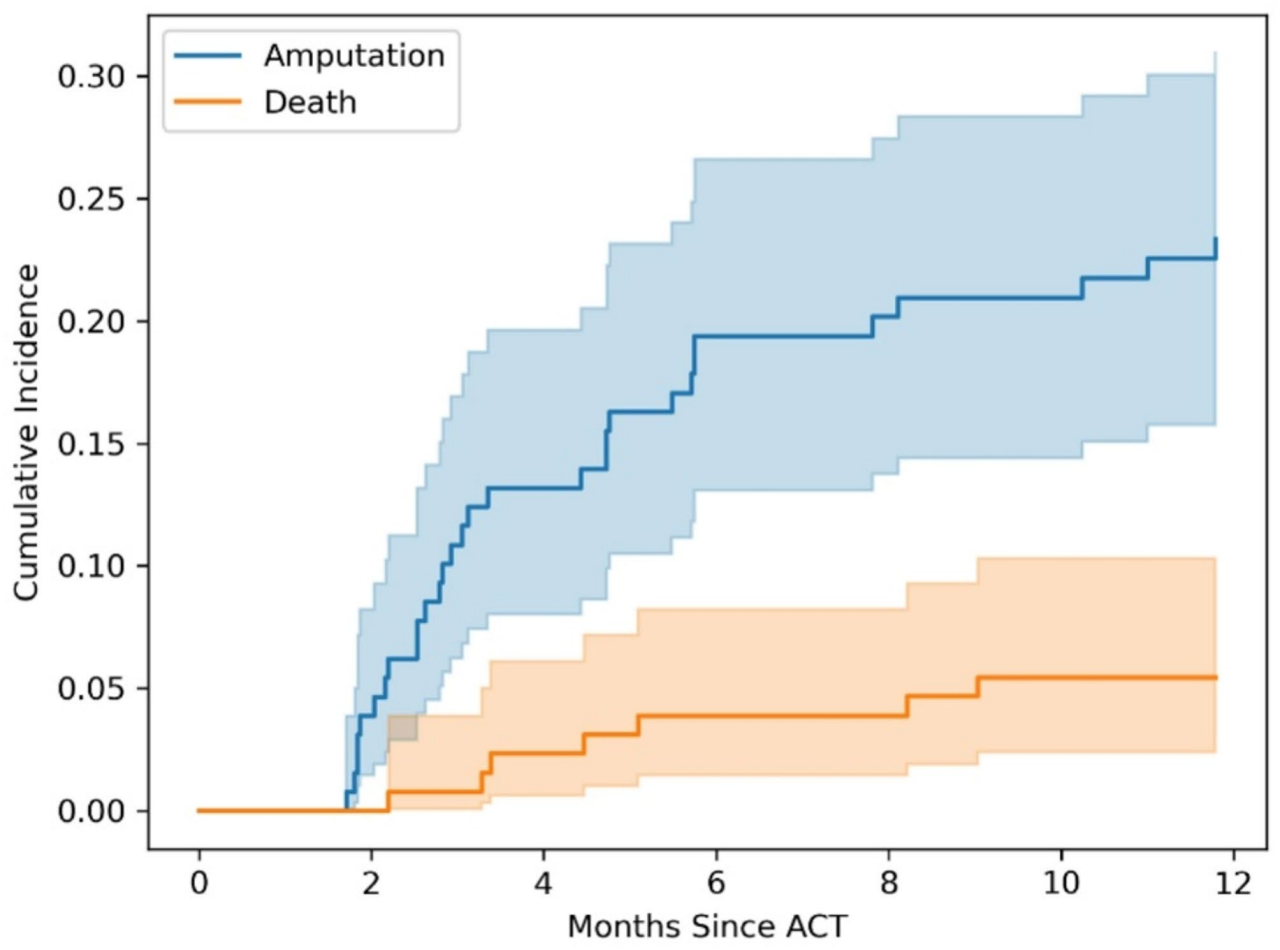
Most of the major amputations occurred at the beginning of the follow-up and more specifically during the first year. In contrast, mortality was spread across the whole follow-up period

During the 15 years from the start of the study we have provided 129 treatments for118 limbs to 107 CLTI patients (Table 1). The total number of treatments is higher than the number of limbs due to re-inclusion of the patient in the ACT study according to the study protocol. Major amputations were performed in 41 limbs (31.8%) in the 16 years follow up. Below-knee amputations were performed in 35 patients and above-knee amputation in 6 patients. The most common reason for major amputation was infection (*n* = 22), followed by ischemia (*n* = 18) and one patient underwent amputation because of ischemic pain. Most of the major amputations (30/41) occurred within the first year after stem cell administration, and predominantly within the first 3 months. The median follow-up duration for participants was 1.92 (0.48–4.61) years, and the median time to a major amputation procedure was 145 (80–406) days. The most common reason for discontinuing follow-up among the three predefined reasons (death, major amputation or new ACT) was death (52 cases). Seven patients (6.5%) died during the first year, and 38 patients (35.5%) during the five years after stem cell treatment. Figure 1 shows the cumulative incidence of major amputations and deaths over 16 years of follow-up and Fig. 2 demonstrates this incidence within the first year after ACT.

**Fig. 3:**
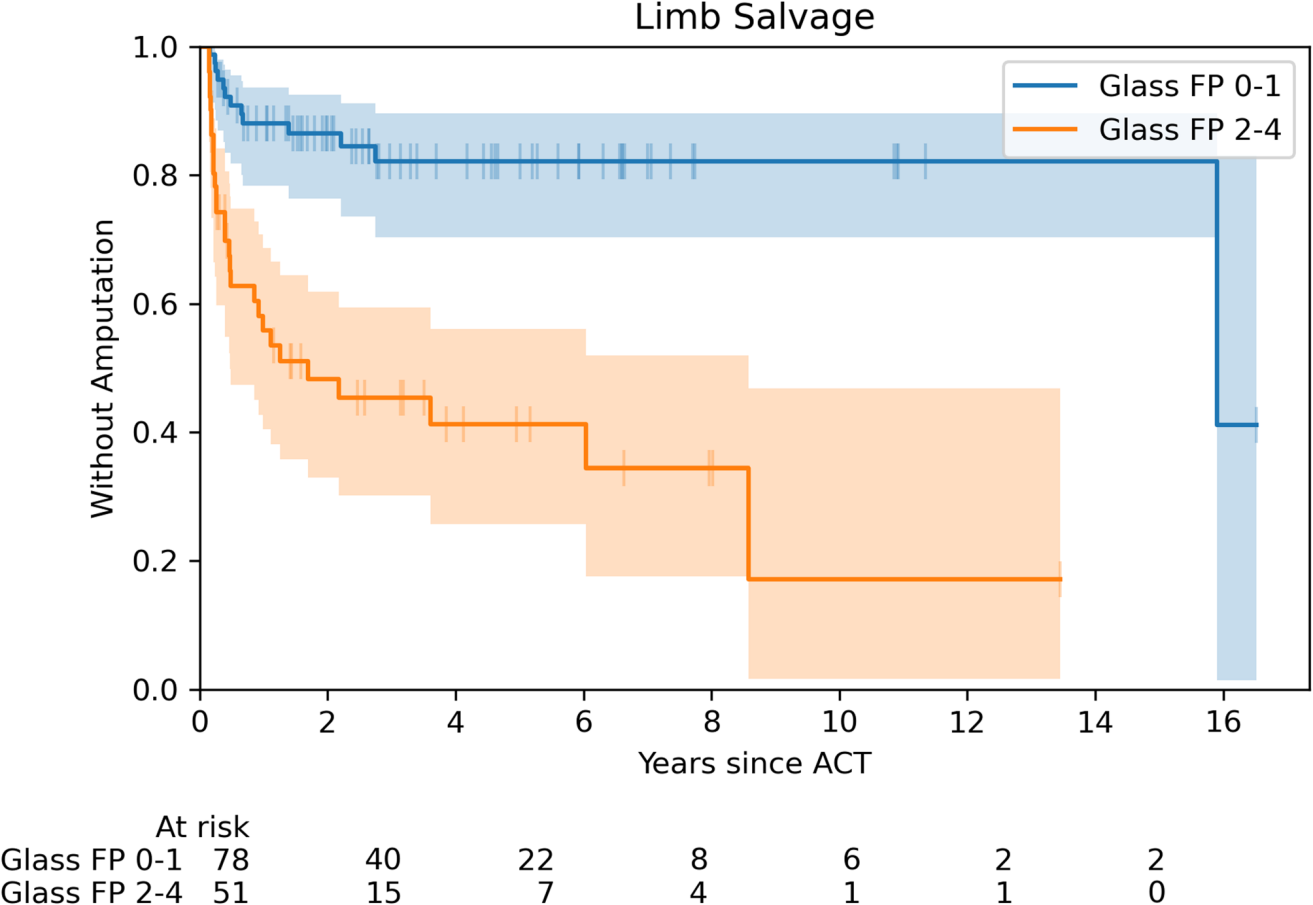
Severe ischemia described by higher scores of GLASS FP classification correlated with shorter follow-up without major amputation after ACT

We assessed the association of the baseline factors with LS and AFS using Cox proportional hazard models in both univariable (Table 2) and multivariable analysis (Table 3). In a univariable analysis, the use of immunosuppressive therapy showed a significant impact on LS after ACT (HR 2.48, CI 1.30–4.73), while the risk is reduced by the use of direct oral anticoagulants (DOACs) (HR 0.16, CI 0.03–0.90). Regarding infection parameters, we observed a significant association between LS and higher grades of WIfI infection score (HR 1.69, CI 1.20–2.38), WIfI composite scores (HR 1.30, CI 1.04–1.62), and higher value of CRP (HR 1.02, CI 1.01–1.03). More severe ischemia described by higher scores of Graziani classification (HR 1.42, CI 1.01–1.99) and GLASS FP (HR 1.58, CI 1.31–1.90), as is shown in Fig. 3, correlated with shorter follow-up without major amputation after ACT. In a multivariable model, only clinical signs of infection (HR 3.21, CI 1.66–6.21) were associated with a higher major amputation rate.

**Table 2:** Results of the effect of baseline parameters on LS and AFS after ACT in univariable analysis

		Limb salavage		Amputation-free survival	
Group	Baseline predictor	HR (95% CI)	*p*-value	HR (95% CI)	*p*-value
Demography	Female	0.54 (0.17, 1.69)	0.29	1.22 (0.70, 2.14)	0.48
	Age [years]	0.97 (0.94, 1.00)	0.048	0.99 (0.97, 1.01)	0.49
	Smoking_ne	0.72 (0.38, 1.36)	0.31	0.79 (0.52, 1.19)	0.26
	Smoking_ano	0.53 (0.18, 1.56)	0.25	0.72 (0.39, 1.35)	0.3
	Smoking_ex	1.75 (0.95, 3.22)	0.073	1.48 (0.96, 2.28)	0.078
Diabetes	DmType_1	0.85 (0.30, 2.43)	0.76	1.08 (0.58, 1.98)	0.81
	DmType_2	0.81 (0.37, 1.78)	0.61	0.81 (0.48, 1.35)	0.41
	DmType_secondary	2.26 (0.96, 5.30)	0.061	1.68 (0.84, 3.34)	0.139
	DmDuration [years]	1.01 (0.98, 1.04)	0.6	1.00 (0.98, 1.02)	0.83
	HbA1c [mmol/mol]	1.02 (1.00, 1.04)	0.095	1.01 (0.99, 1.02)	0.25
	DmTherapy_Insulin	1.48 (0.68, 3.19)	0.32	1.15 (0.76, 1.74)	0.51
	DmTherapy_OAD	0.40 (0.14, 1.16)	0.092	0.74 (0.48, 1.16)	0.189
	DmTherapy_Tx	1.86 (0.43, 7.97)	0.4	1.49 (0.48, 4.64)	0.49
	DmTherapy_dieta	1.96 (0.70, 5.47)	0.199	1.50 (0.65, 3.45)	0.34
Complications	CKD	0.85 (0.66, 1.08)	0.186	**1.25 (1.08**,** 1.43)**	**0.002**
	HD	0.77 (0.33, 1.81)	0.55	**2.05 (1.33**,** 3.16)**	**0.001**
	EyeComplications	0.85 (0.45, 1.57)	0.59	0.93 (0.61, 1.41)	0.72
	Neuropathy	0.86 (0.41, 1.80)	0.69	0.85 (0.52, 1.40)	0.53
	CAD	0.83 (0.42, 1.62)	0.58	1.33 (0.81, 2.17)	0.26
	HeartFailure	1.36 (0.73, 2.52)	0.33	1.28 (0.83, 1.97)	0.26
Nutrition and lipids	BMI [kg/m2]	1.02 (0.95, 1.09)	0.57	1.02 (0.97, 1.07)	0.5
	Albumin [g/L]	1.00 (0.94, 1.08)	0.94	**0.93 (0.89**,** 0.98)**	**0.002**
	TAG [mmol/L]	1.17 (0.89, 1.54)	0.26	1.06 (0.86, 1.30)	0.57
	Cholesterol [mmol/L]	0.94 (0.69, 1.28)	0.71	1.03 (0.85, 1.26)	0.75
	LDL [mmol/L]	0.90 (0.62, 1.31)	0.58	1.03 (0.82, 1.29)	0.8
Medication	Immunosuppression	**2.48 (1.30**,** 4.73)**	**0.006**	**1.81 (1.10**,** 2.96)**	**0.019**
	Statins	1.59 (0.83, 3.02)	0.16	0.97 (0.64, 1.46)	0.88
	LMWH	1.08 (0.40, 2.94)	0.88	1.43 (0.74, 2.75)	0.29
	P2Y12 inhibitors	0.91 (0.44, 1.90)	0.81	0.97 (0.59, 1.59)	0.9
	ASA	1.00 (0.52, 1.90)	0.99	0.79 (0.53, 1.19)	0.26
	Warfarin	1.45 (0.62, 3.40)	0.39	1.22 (0.65, 2.27)	0.53
	DOACs	**0.16 (0.03**,** 0.90)**	**0.038**	0.72 (0.44, 1.16)	0.179
Ischemia	TcPO_2_ [mmHg]	0.97 (0.95, 1.00)	0.025	0.99 (0.98, 1.01)	0.47
	Rutherford	1.70 (0.90, 3.20)	0.1	1.01 (0.59, 1.71)	0.98
	Graziani	**1.42 (1.01**,** 1.99)**	**0.044**	**1.31 (1.04**,** 1.65)**	**0.022**
	Glass FP	**1.58 (1.31**,** 1.90)**	**< 0.001**	**1.28 (1.13**,** 1.46)**	**< 0.001**
	Glass IP	0.90 (0.54, 1.51)	0.69	1.05 (0.71, 1.56)	0.79
	Glass P	1.17 (0.66, 2.08)	0.58	1.27 (0.88, 1.82)	0.2
DFU classification	Wagner	1.21 (0.88, 1.68)	0.24	1.04 (0.85, 1.27)	0.71
	Wifi Wound	0.93 (0.56, 1.56)	0.79	1.03 (0.74, 1.43)	0.85
	Wifi Ischemia	1.37 (0.49, 3.78)	0.55	1.61 (0.72, 3.57)	0.25
	Wifi Infection	**1.69 (1.20**,** 2.38)**	**0.003**	1.28 (0.99, 1.64)	0.057
	Wifi Composite	**1.30 (1.04**,** 1.62)**	**0.023**	1.16 (0.99, 1.36)	0.07
	Sinbad	0.92 (0.69, 1.23)	0.57	1.06 (0.88, 1.29)	0.54
	TexasGrade_C	1.20 (0.69, 2.06)	0.52	1.23 (0.85, 1.79)	0.27
	TexasStage_D	0.82 (0.42, 1.63)	0.58	0.95 (0.60, 1.50)	0.82
Infection	Clinical infection	**3.21 (1.66**,** 6.21)**	**< 0.001**	**1.99 (1.26**,** 3.15)**	**0.003**
	OM X-ray signs	0.77 (0.39, 1.52)	0.46	0.71 (0.45, 1.13)	0.147
	MRSA	2.08 (0.98, 4.45)	0.058	1.33 (0.65, 2.70)	0.43
	Resistant bacteria	1.26 (0.68, 2.34)	0.47	**1.56 (1.04**,** 2.33)**	**0.031**
	CRP [mg/l]	**1.02 (1.01**,** 1.03)**	**< 0.001**	1.01 (1.00, 1.02)	0.22
Isolation method	Gelofusin	1.16 (0.58, 2.31)	0.68	0.93 (0.58, 1.48)	0.75
Final product	Leukocytes [10^9/L]	1.01 (1.00, 1.01)	0.26	1.01 (1.00, 1.01)	0.129
	Lymphocytes [10^9/L]	1.02 (0.99, 1.06)	0.23	0.99 (0.96, 1.03)	0.76
	Monocytes [10^9/L]	1.01 (0.74, 1.38)	0.94	1.04 (0.86, 1.26)	0.69
	Thrombocytes [10^9/L]	1.00 (1.00, 1.00)	0.48	1.00 (1.00, 1.00)	0.4
	CD34pct [%]	0.71 (0.27, 1.85)	0.49	0.63 (0.31, 1.28)	0.2
	CD34abs [10^6/l]	1.00 (1.00, 1.00)	0.97	1.00 (1.00, 1.00)	0.68
	Viability [%]	0.99 (0.98, 1.01)	0.33	1.00 (0.99, 1.00)	0.55

Abbreviation:

OAD - oral antidiabetic drugs, CKD - chornic kidney disease, HD– hemodialysis, CAD - coronary artery disease, BMI - body mass index, ASA– aspirin, LMWH - low-molecular-weight heparin, DOAC - direct oral anticoagulants, TcPO2 - transcutaneous oxygen pressure, GLASS - Global Limb Anatomic Staging System, FP - femoropopliteal segment, IP - infrapopliteal segment, P - pedal segment, OM– osteomyelitis, MRSA - Methicillin-resistant Staphylococcus aureus

**Table 3:** Results of correlation between baseline parameters and LS and AFS in multivariable models

		Limb salvage		Amputaion-free survival	
Group	Baseline predictor	HR (95% CI)	*p*-value	HR (95% CI)	*p*-value
Demography	Female	0.765 (0.132, 4.445)	0.765	1.427 (0.729, 2.793)	0.300
	Age [years]	0.972 (0.942, 1.003)	0.075	1.008 (0.985, 1.032)	0.493
Diabetes	Duration [years]	1.016 (0.984, 1.049)	0.322	1.002 (0.978, 1.026)	0.890
	HbA1c [mmol/mol]	1.016 (0.992, 1.041)	0.200	1.015 (0.997, 1.033)	0.103
Complications	CKD	0.837 (0.583, 1.202)	0.335	1.067 (0.877, 1.299)	0.515
Medications	Immunosuppression	2.683 (0.961, 7.489)	0.059	**2.318 (1.085**,** 4.952)**	**0.030**
Ischemia	TcPO_2_ [mmHg]	0.998 (0.969, 1.027)	0.871	**1.025 (1.003**,** 1.048)**	**0.026**
DFU classification	WifiComposite	1.135 (0.825, 1.561)	0.438	**1.252 (1.019**,** 1.539)**	**0.033**
Infection	Clinical infection	**2.211 (1.010**,** 4.839)**	**0.047**	1.569 (0.916, 2.688)	0.101
	OM X-ray signs	0.878 (0.397, 1.943)	0.748	0.818 (0.484, 1.382)	0.453
Final product	Leukocytes [10^9/L]	1.002 (0.989, 1.014)	0.812	1.002 (0.992, 1.011)	0.729
	CD34_percentage [%]	1.640 (0.355, 7.579)	0.526	1.091 (0.413, 2.881)	0.860

Abbreviations:

CKD - chronic kidney disease, TcPO2 - transcutaneous oxygen pressure, OM - osteomyelitis

With regards to AFS, patients with a higher grade (stage 4 or 5) of chronic kidney disease (HR 1.25, CI 1.08–1.43), and receiving hemodialysis (HR 2.05, CI 1.33–3.16) had a higher risk of amputation and death. On the other hand, higher blood albumin level was associated with reduced risk (HR 0.93, CI 0.89–0.98). Presence of clinical signs of local infection (HR 3.21, CI 1.66–6.21), and the evidence of bacteria resistant to peroral antibiotics in the wound (HR 1.56, CI 1.04–2.33) were significantly associated with AFS in univariable analysis. The most common organism isolated from the wound was Pseudomonas aeruginosa (P. aeruginosa) (*n* = 23) followed by Enterococcus faecalis and aerogenes (*n* = 17).

**Fig. 4:**
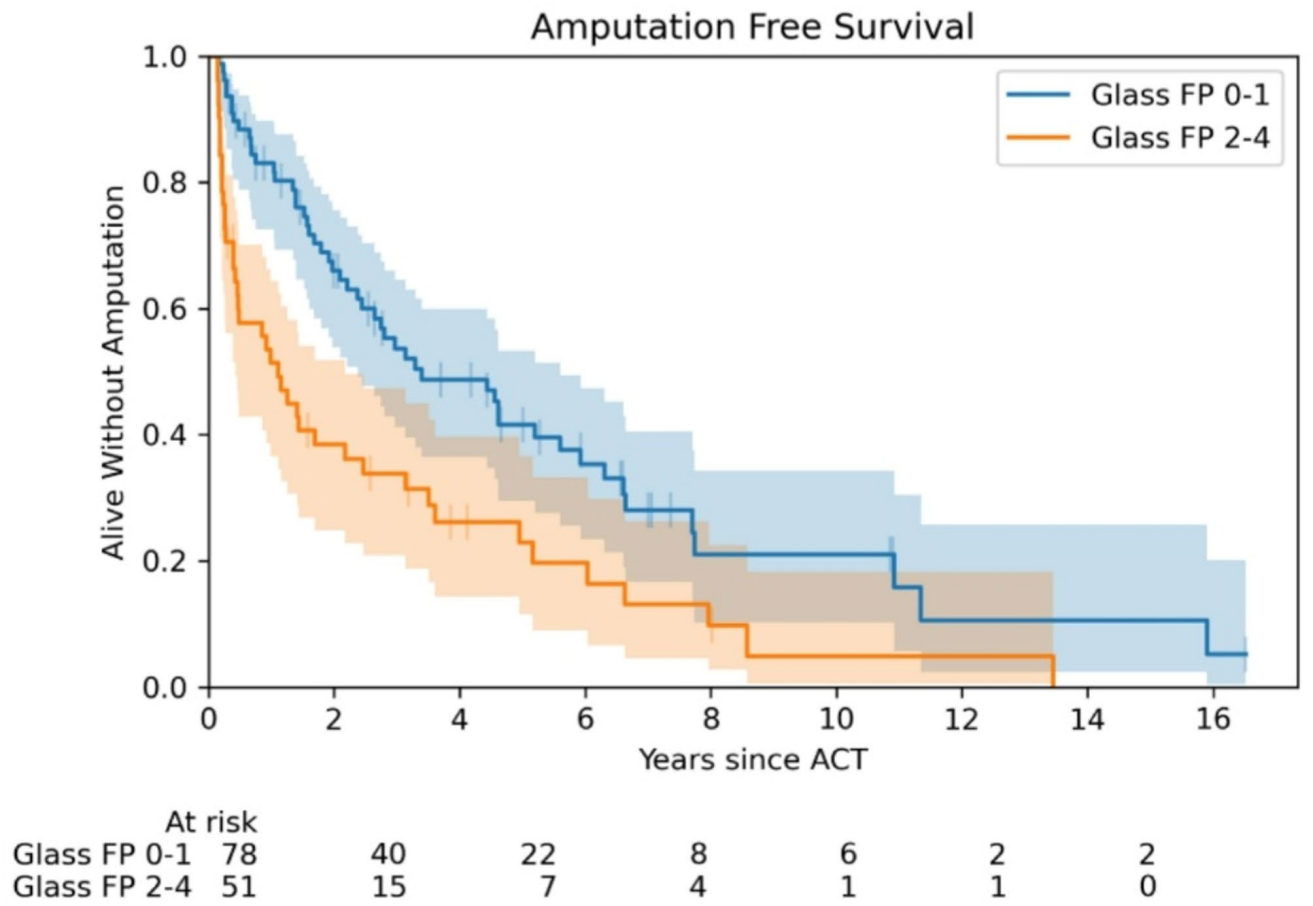
Patients with higher stages of GLASS FP, i.e. more extensive stenosis of the FP segment, had a higher risk of amputation or death

As in LS, Graziani classification (HR 1.31, CI 1.04–1.65) and Glass FP (HR 1.28, CI 1.28–1.46), were associated with shorter AFS (Fig. 4). In a multivariable model, we found that the use of immunosuppressive therapy (HR 2.32, CI 1.09–4.95), lower values of TcPO_2_ (HR 1.03, CI 1.00–1.05), and higher grades of WIfI composite score (HR 1.25, CI 1.02–1.54) at baseline, were linked to worse AFS.

## Discussion

This is the first study to report such long follow-up in patients receiving ACT. The rate of major amputation after ACT was 34.8% during 16 years of follow-up with the highest frequency in the first year (23.3%). In retrospective analysis of prospectively collected data from the JUVENTAS randomized controlled trial assessing stem cell therapy, 33% of patients during 5 years after therapy and amputations occurred predominantly in the first year. We must note that there are also some differences, because patients in the JUVENTAS study were treated by BM-MNCs via intra-arterial infusion and were included only by ABI index which could be influenced by medial sclerosis of lower limb arteries [18]. For comparison no-option CLTI patients without any revascularization treatment have a 30% risk of major amputation within the first year vs. 4.5% in patients with possible revascularization [19]. Meta-analyses, focusing on ACT therapy, showed that ACT is more effective than standard therapy in the reduction of major amputations [20, 21]. These meta-analyses also described the impact of stem cell therapy on ulcer healing, improved ischemia parameters, rest pain, and extending the walking distance without claudication pain. On the other hand, in the recent PACE randomized clinical trial which evaluated PLX-PAD (PLacental eXpanded) therapy in CLTI patients versus placebo, similar AFS rates were observed between the two groups [9]. In contrast with our study, the PACE study used mesenchymal allogeneic cells in ACT and included patients both with and without diabetes.

The mortality of the patients in our study was 6.5% in the first year and 35.5% in five years after ACT, which is explained by the polymorbidity of enrolled patients. No-option CLTI patients have a high mortality rate with a mortality risk of around 20–25% within one year and approximately 60% within five years after CLTI diagnosis [5]. In our cohort, while the mortality was spread across the whole follow-up period, most of the amputations happened during the first year. So the first year is the most important time for the clinical effect of ACT to manifest, and also the most risky period for the occurrence of phlegmon or worsening ischemia, or pain for which the patient might undergo major amputation.

Patients taking DOACs as chronic medication avoided major amputation for a longer period after ACT. There is no direct evidence that they enhance the benefits of ACT directly, but there are few published studies about the benefits of DOAC to angiogenesis. Rivaroxaban improved blood flow recovery and increased capillary density in streptozotocin-induced diabetic mice with limb ischemia. Mechanisms included upregulation of VEGF and endothelial nitric oxide synthase, critical for angiogenesis. Moreover, it reduced factor Xa-mediated inflammation (e.g., IL-6, IL-8, CCL2) and endothelial permeability, preserving vascular function [22, 23]. Apixaban reversed endothelial damage in human microvascular endothelial cells exposed to uremic serum, so it might maintain vascular function in chronic kidney disease, indirectly supporting angiogenesis. Edoxaban did not directly promote angiogenesis but preserved endothelial health and mitigated factor Xa-driven anti-angiogenic pathways. Dabigatran also showed anti-inflammatory effects, whereas factor Xa inhibitors do not have this function [24–27].

We observed a significant association of clinical signs of infection, higher stages of WIfI infection score, and higher levels of CRP values in a univariable analysis on LS. Moreover, the negative effect of clinical infection was confirmed by multivariable analysis. Thus, it can be assumed that infection was the main part of the WIfI composite score that made it significant. In general, elevated CRP is a marker of infection which significantly impairs wound healing in diabetic patients with CLTI [28]. We have already described the association between higher CRP levels and the risk of major amputation after ACT in a previous paper [29]. Other inflammation markers associated with poor prognosis after cell therapy include leukocytosis, lower levels of IL-10 (anti-inflammatory cytokine enhancing tissue repair), and higher levels of CD45 + inflammatory cells infiltrated in the wound [30, 31]. We also demonstrated that a higher WIfI composite score shortens AFS. For the significance of clinical signs of infection, we can assume that WIfI infection is the component with the highest proportion of AFS effect. In conclusion, the inflammatory status of the patient at the time of stem cell administration is strongly related to the risk of major amputations.

In contrast to most published studies, we also included immunosuppressed patients from various conditions who were mainly treated with tacrolimus, mycophenolate mofetil, prednisone, and their combinations. Immunosuppressive therapy correlated with major amputations and the significance of AFS was even confirmed in the multivariable model. It is known that immunosuppressants like calcineurin inhibitors (cyclosporine, tacrolimus) and mTOR inhibitors (sirolimus, everolimus) can suppress VEGF signalling, reducing new blood vessel formation in ischemic tissues, which could have had an impact on worsening circulation and hence increased amputation risk [32, 33]. Except for suppressing the inflammatory response, potentially inhibiting the natural repair processes involved in vascular remodelling, they can reduce endothelial function, promoting vascular stiffness, and impairing the proliferation of vascular smooth muscle cells [34–36].

This is the first study which analysed GLASS, with the efficacy of ACT. The higher score of GLASS FP was a risk factor for both LS and AFS. However, this result was only observed in univariable analysis and this classification, which is primarily focused on macrocirculation, is necessary for ACT to be used in combination with other microcirculation tests (e.g. TcPO_2_). The risk predictor for LS was seen in the higher score in the Graziani anatomical classification. A study assessing Graziani’s classification for the success of percutaneous intervention found that stage 1–2 (less severe disease) correlated with higher rates of technical revascularization success compared to stages 3–5 [37]. In the multivariable model, a lower value of TcPO_2_ was the negative predictor for AFS. Ulcer healing and limb prognosis are in general poor if TcPO_2_ is < 20 mm Hg and better if TcPO_2_ is > 40 mm Hg; however, even these values varied between studies [38].

Although the rate of major amputations after standard methods of revascularization varies by age (patients ≤ 50 years with premature PAD face the highest rate of major amputations due to aggressive disease progression and early comorbidities), we did not demonstrate a significant difference in our study [39]. However, our results confirmed that severe stages of CKD (stages 4 and 5) and hemodialysis treatment reduce AFS, but not LS. Thus, these risk factors are connected most likely with the polymorbidity of patients rather than ACT itself. Patients with both CLTI and CKD, especially with advanced or end-stage kidney disease (ESRD), have a poor prognosis even after standard revascularization. Patients with ESRD who underwent infrapopliteal angioplasty had twice the risk of wound healing failure, re-angiopasty, and death or major amputation than those without ESRD [40].

Hypoalbuminemia is a known risk factor for poor DFU healing and it was not studied in the context of ACT [41, 42]. But it was associated with higher mortality and perioperative morbidity in patients who underwent infrapopliteal bypass [43]. Therefore, poor nutrition, as measured by albumin, could have a negative impact on the mortality of these patients, but other parameters of nutrition (BMI, cholinesterase or triacylglycerols) were not significant.

The presence of resistant bacteria was a significant risk factor for AFS. Infections caused by resistant pathogens, like Enterobacteriaceae and Pseudomonas aeruginosa are linked to increased mortality and healthcare costs worldwide [44]. Enterobacteriaceae (particularly Klebsiella spp., Proteus spp., and Morganella spp.) are strongly linked to higher rates of major amputation in DFU [45]. Biofilms formed by K. pneumoniae reduce antibiotic efficacy and prolong time in inflammation, delaying healing [46]. Surprisingly, P. aeruginosa produces elastase and exotoxins that degrade tissue but do not independently delay healing in well-managed ulcers [47].

Parameters related to diabetes, such as type, duration and others, was not significant either for LS or AFS, but we used only HbA1c for assessment of glycemic control in contrast to PACE study. However, for a better understanding of the relationship between glycaemic control and ACT success, it is needed to involve parameters from the glucose sensor (e.g. time in range, time above and below range).

We didn’t observe any significant impact of final cell product parameters on LS or AFS. However, we analysed only the CD34 subpopulation. There are already several other subpopulations described which may play a role in this therapy and could influence its efficacy, like CD34 + CD133+, CD34 + CD133 + CD309 + and others which we plan to assess in the future research [48, 49].

This study highlights the results of autologous cell therapy in relation to baseline parameters, up to 16-years of follow-up. Our study was unique in that dialysis patients, as well as patients who were treated with immunosuppression, were included in our study, whereas in most other studies, they were excluded. The main difference in the design of our study in comparison to others like PROVASA [50], JUVENTAS [51], RESORE-CLI [52] or PACE [9] is the absence of a control group, which is its main limitation. There is a limited number of studies which discuss factors influencing the results of cell therapy. The conclusion of parameters for responders for cell therapy published in different studies is written in Table 4. However, these studies included heterogeneous groups of people and used different types of cells and methods. Among these parameters, we confirmed the influence of chronic kidney disease and inflammatory state.

**Table 4:** Characteristics of respondents described in previous studies

Baseline parameter	Association with better response
Age	Younger patients (< 50 years) 29
Smoking status	Non-smokers or ex-smokers (28)
Comorbidities	Absence of diabetes, hypertension, or renal dysfunction (29)
Diabetes	Well-controlled (HbA1c < 6.5%) (9)
Inflammatory markers	Lower baseline CRP, IL-6, and leukocyte counts (28, 29)
Cell product quality	Higher CD34 + cell counts (> 10^6^ cells/kg) and viability (29)
Thrombophilia genetic mutations	Absence of MTHFR mutation, Leiden mutation and normal protein C level (28, 53)

Allogeneic cells as ACT may be more efficacious and has been clinically demonstrated previously. A recent randomized double-blind study observed that patients treated with the allogeneic Wharton jelly-derived MSCs showed faster clinical improvement compared to autologous MB-MNCs without the need for immunosuppression, and without any adverse events, including immune reactions. The main limitation was the small number of patients and larger studies are needed [54]. Another method for better success of therapy is to choose the most effective route of administration. Intramuscular injection of precursor cells offers the advantage of injecting cells close to the ulcer, which stimulates paracrine activity and the release of angiogenic cytokines such as VEGF (vascular endothelial growth factor), bFGF (basic fibroblast growth factor) and others. In contrast, intra-arterial administration may improve systemic perfusion but increases the risk associated with the potential arterial injury. Moreover, Klepanec et al. found no significant difference in limb salvage or ulcer healing between intramuscular and intra-arterial administration [55]. Finally, a lot of research is focused on the modification of cells through preconditioning, genetic modification, and biomaterial scaffolds to enhance therapeutic outcomes. Hypoxic preconditioning activates hypoxia-inducible transcription factor-1α (HIF-1α) and pro-survival pathways, increasing resistance to oxidative stress and apoptosis [56]. Overexpressing angiogenic factors like VEGF and green fluorescent protein (eGFP) improved limb reperfusion and muscle repair effects. Surface receptor engineering, including E-selectin-coated MSCs, enhances targeted homing to ischemic tissues, potentiates angiogenesis and wound repair. Hydrogel encapsulation allows for sustained release of growth factors improving MSC retention and arteriogenesis [57].

## Limitations of the study

The main limitation of this study was the absence of a control group; only patients treated by ACT were included. This was not possible because it was considered unethical to provide bone marrow harvesting in polymorbid patients as well as use intramuscular saline injections in patients with no-option CLTI. We are fully appreciative that placebo-controlled design minimizes the risk of bias, ensures that both groups receive identical care in terms of procedures, and provides valuable data on the efficacy of the cell therapy. The way to create this type of study is to design the study carefully to balance scientific rigor with patient welfare - careful informed consent, transparent patient communication, frequent monitoring, and post-trial access to effective treatments. Sham injection must be low-risk and does not expose patients to unnecessary harm. All participants would be closely monitored for adverse events or complications, including both the experimental and placebo groups. Stopping rules must be defined for the trial, where it can be terminated early if significant harm is detected or if it is evident that the experimental therapy is overwhelmingly superior to the placebo.

To be more specific, the main decision would be the choice of the type of stem cells administered (BM-MNCs, PM-MNCs, MSCs or eventually others). This decision determines the invasiveness of harvesting. Among the cells with the least invasive harvesting are MSCs that would be collected from adipose tissue, even from another type of tissue, which is a great advantage of these cells.

In this way, the risk of harvesting can be reduced, but the risk from injecting cells into the muscle of the ischemic limb remains. A way to at least reduce this risk is to research specific injection locations from which to maximize the effectiveness of the cells. Since stenoses are variable from patient to patient, these locations would have to be modified for each individual person.

During the study, we had to use 2 different methods of bone marrow separation due to technical circumstances (Harvest separation and Gelofusin separation), so the processing was not done in the same way in all patients. However, according to the outcomes, there was no difference between these two methods.

## Conclusion

This study presented ACT as a prospective method of revascularization for no-option CLTI patients. Our results suggest that the best initial clinical status of the patient at the time of stem cell administration is the absence of any signs of infection, and stenosis of FP segments. ACT therapy should be more strictly considered in patients with the presence of severe stages of CKD, hemodialysis and those treated with immunosuppressive therapy. Because most of the amputations have been performed within the first year after the therapy, it is the most important period for reducing major amputation rates and prolonging AFS by close follow-up and aggressive management of risk factors such as infection and poor nutritional status.

## Data Availability

The dataset with analysis methods used in the study is available at [10.5281/zenodo.15111180] [56].

## Declarations

### Artificial intelligents (AI)

The authors declare that they have not use AI-generated work in this manuscript.
